# Methodological considerations regarding Sigma-1 receptor activation and autophagic flux in renal ischemia-reperfusion injury

**DOI:** 10.1080/0886022X.2026.2757311

**Published:** 2026-11-09

**Authors:** Sooraj Kumar

**Affiliations:** Department of Medicine, Liaquat University of Medical and Health Sciences (LUMHS), Jamshoro, Sindh, Pakistan

To the Editor

We read with great interest the recent study by Gong et al. investigating the renoprotective effects of the Sigma-1 receptor (σ_1_R) in ischemia-reperfusion injury (IRI) [1]. The authors present compelling data suggesting that fluvoxamine-mediated σ_1_R activation preserves mitochondrial function, reduces reactive oxygen species, and attenuates cellular apoptosis [1]. However, we respectfully highlight two methodological limitations regarding pharmacological specificity and autophagic flux that warrant further discussion.

First, the *in vivo* model relies exclusively on fluvoxamine (20 mg/kg) administered prior to ischemia to demonstrate σ_1_R-mediated renoprotection [1]. While fluvoxamine acts as a σ_1_R agonist, it is primarily a selective serotonin reuptake inhibitor (SSRI) with well-documented systemic and pleiotropic effects. In robust pharmacological studies, attributing *in vivo* outcomes specifically to σ_1_R requires the co-administration of a highly specific σ_1_R antagonist, such as BD1047 or BD1063, or the utilization of a Sigmar1 knockout murine model [3]. Because the authors did not utilize a specific antagonist to block fluvoxamine’s protective effects, the attenuation of renal injury cannot be definitively isolated to σ_1_R agonism, as it may be confounded by the off-target serotonergic or systemic anti-inflammatory properties of the drug [1,3].

Second, the *in vitro* assays assess mitophagy using static markers (LC3B-II, p62, TOM20, and PINK1/Parkin) alongside mitochondria-lysosome colocalization *via* fluorescent tracking [1]. According to the universally recognized guidelines for monitoring autophagy established by Klionsky et al. static markers alone are insufficient to distinguish between enhanced autophagic flux and a pathological blockade of autophagosome-lysosome degradation, particularly in acute stress models like ischemia [2]. Renal IRI is known to severely impair lysosomal membrane integrity and acidification. Therefore, without utilizing late-stage autophagic flux inhibitors, such as bafilomycin A1, an increase in LC3B-II and colocalization could technically represent a toxic accumulation of undegraded autophagosomes rather than successful mitophagic clearance [1,2].

We commend Gong et al. for their valuable contribution to the study of acute kidney injury [1]. Clarifying these pharmacological and biochemical dynamics in future studies will further solidify σ_1_R as a reliable therapeutic target in nephrology [1,3].

## Data Availability

Data sharing is not applicable to this article as no new primary data were created or analyzed in this correspondence.
